# Predicting Pathological Features at Radical Prostatectomy in Patients with Prostate Cancer Eligible for Active Surveillance by Multiparametric Magnetic Resonance Imaging

**DOI:** 10.1371/journal.pone.0139696

**Published:** 2015-10-07

**Authors:** Ottavio de Cobelli, Daniela Terracciano, Elena Tagliabue, Sara Raimondi, Danilo Bottero, Antonio Cioffi, Barbara Jereczek-Fossa, Giuseppe Petralia, Giovanni Cordima, Gilberto Laurino Almeida, Giuseppe Lucarelli, Carlo Buonerba, Deliu Victor Matei, Giuseppe Renne, Giuseppe Di Lorenzo, Matteo Ferro

**Affiliations:** 1 Division of Urology, European Institute of Oncology, Milan, Italy; 2 Department of Translational Medical Sciences, University “Federico II”, Naples, Italy; 3 Division of Epidemiology and Biostatistics, European Institute of Oncology, Milan, Italy; 4 Department of Radiotherapy, European Institute of Oncology, Milan, Italy; 5 Department of Radiology, European Institute of Oncology, Milan, Italy; 6 University of Vale do Itajaí, Catarinense Institute of Urology, Division of Laparoscopy, Itajaí, Brazil; 7 Department of Emergency and Organ Transplantation, Urology and Kidney Transplantation Unit, University of Bari, Bari, Italy; 8 Division of Medical Oncology, CROB—IRCCS, Rionero in Vulture, Italy; 9 Division of Pathology, European Institute of Oncology, Milan, Italy; 10 Medical Oncology Unit, Department of Clinical Medicine, Federico II University, Naples, Italy; Cedars Sinai Medical Center, UNITED STATES

## Abstract

**Purpose:**

The aim of this study was to investigate the prognostic performance of multiparametric magnetic resonance imaging (mpMRI) and Prostate Imaging Reporting and Data System (PIRADS) score in predicting pathologic features in a cohort of patients eligible for active surveillance who underwent radical prostatectomy.

**Methods:**

A total of 223 patients who fulfilled the criteria for “Prostate Cancer Research International: Active Surveillance”, were included. Mp–1.5 Tesla MRI examination staging with endorectal coil was performed at least 6–8 weeks after TRUS-guided biopsy. In all patients, the likelihood of the presence of cancer was assigned using PIRADS score between 1 and 5. Outcomes of interest were: Gleason score upgrading, extra capsular extension (ECE), unfavorable prognosis (occurrence of both upgrading and ECE), large tumor volume (≥0.5ml), and seminal vesicle invasion (SVI). Receiver Operating Characteristic (ROC) curves and Decision Curve Analyses (DCA) were performed for models with and without inclusion of PIRADS score.

**Results:**

Multivariate analysis demonstrated the association of PIRADS score with upgrading (P<0.0001), ECE (P<0.0001), unfavorable prognosis (P<0.0001), and large tumor volume (P = 0.002). ROC curves and DCA showed that models including PIRADS score resulted in greater net benefit for almost all the outcomes of interest, with the only exception of SVI.

**Conclusions:**

mpMRI and PIRADS scoring are feasible tools in clinical setting and could be used as decision-support systems for a more accurate selection of patients eligible for AS.

## Introduction

The use of prostate specific antigen (PSA) testing has recently been criticized for prostate cancer (PCa) screening[1,2], although it continues to be the best biomarker available for early PCa detection. The increasing use of this biomarker in association with several PSA derivatives, such as free to total PSA ratio (%fPSA), PSA density (PSAD), and PSA velocity, has led to frequent detection of small, well differentiated, low-risk PCa without significant decrease in mortality[3]. This fact gives rise to the thought that clinically insignificant disease is being treated excessively and active follow up of these patients should be preferred instead of radical treatment. Active surveillance (AS) is an alternative to initial radical treatment of low-risk PCa, even if the current parameters used for selection and follow up, such as clinical T stage, total PSA, PSA density, Gleason score (GS), and number of positive prostate biopsy cores, incorrectly exclude some patients eligible for AS and misclassify some who actually harbor significant disease[4]. In order to predict the pathologic findings at radical prostatectomy, risk stratification has been improved with validation of several nomograms that aid to reduce the rates of overtreatment in patients with clinically insignificant PCa[5]. Consequently numerous preoperative prognostic tools have analyzed the ability of prostate cancer antigen 3 (PCA3), sarcosine, [–2]proPSA, and Prostate Health Index (PHI) in predicting pathological features at radical prostatectomy[6,7]_._ Multiparametric magnetic resonance imaging (mpMRI) is increasingly being used in clinical practice to evaluate PCa localization, tumor stage and aggressiveness aiding treatment planning[8]_._ Although many studies available on the role of mpMRI during PCa-AS have shown the ability to reduce re-biopsies[9,10], not always MRI lesions correspond with guided biopsy or radical prostatectomy (RP) specimen findings[11]. Recently preoperative neural network software including mpMRI variables, PSA level and GS has been reported to predict insignificant prostate cancer, particularly in the context of clinically non-palpable tumors, suggesting a prognostic and pathologic predictive role in clinically very low risk PCa[12]. In this scenario it has been developed a scoring system called Prostate Imaging Reporting and Data System (PIRADS), with the aim to enable elaboration, interpretation, and reporting of prostate mpMRI findings[13]. The aim of this study is to investigate the prognostic performance of MRI and PIRADS score in predicting pathologic features in a cohort of patients eligible for active surveillance who underwent RP.

## Patients and Methods

We retrospectively reviewed the medical records of 2,200 patients who underwent robotic RP for PCa between November 2009 and July 2014. None of the patients included in the current study received neoadjuvant androgen-deprivation therapy or drugs that could alter the PSA values. In total 223 patients fulfilled the inclusion criteria for “Prostate Cancer Research International: Active Surveillance”[14] defined as follows: clinical stage T2a or less, PSA<10 ng/ml, 2 or fewer cores involved with cancer after a 12-core biopsy scheme, GS≤6 grade and PSA density<0.2ng/mL/cc. We compared the pathological findings between prostate biopsies and specimens after RP. Specimens were processed and evaluated according to the Stanford protocol[15] by a single, experienced, genitourinary pathologist(G.R.) blinded to index-tests results. After fixing the RP specimens, they were inked and cut at 3-mm intervals perpendicular to the rectal surface. The apical slice was cut para-sagittally at 2-3-mm intervals, and the sections were then divided in halves or quadrants to fit routinely used cassettes for paraffin embedding. The whole prostate was sampled.

This retrospective analysis of prospectively acquired data was approved by the “IRCCS—Istituto Europeo di Oncologia Ethic Committee” who waived the requirement for informed consent specific to the study because all patients provided written informed consent for MR imaging, surgical procedures, and research use of their medical information.

Mp–1.5 Tesla MRI (Avanto; Siemens Medical Solutions, Erlangen, Germany), examination staging with endorectal coil was performed at least 6–8 weeks after TRUS-guided biopsy, in order to avoid distortions and artifacts due to inflammatory process after the bioptic procedure. The following pulse sequences were used: sagittal, coronal, and axial T2-TSE (TR/TE, 831/80 ms), axial Diffusion-Weighted Imaging (DWI) using high b values (b = 800) and ADC maps, axial Dynamic Contrast-Enhanced imaging (DCE) obtained before, during and after injection of gadopentetate dimeglumine (Magnevist; Bayer Healthcare, Berlin, Germany) administered at a dose of 0.1 mmol per kilogram of body weight through a peripheral vein at a flow rate of 3 mL/sec followed by a saline bolus of 10 mL administered at the same flow rate by using a mechanical injector (Spectris MR Injection System; Medrad, Leverkusen, Germany) and axial T1-TSE (TR/TE, 217.8/4.6).

The European Society of Urogenital Radiology (ESUR) in 2012 established clinical guidelines for the acquisition, interpretation, and reporting of mpMRI of the prostate in order to facilitate a greater level of standardisation and consistency[16]. These recommendations, popularly referred to as Prostate Imaging Reporting and Data System (PI-RADS), were based on literature evidence and consensus expert opinion.

One radiologist (G.P.) prospectively read and scored all cases, developing a standardized structured report for each patient. In all patients, the likelihood of the presence of cancer was assigned using PIRADS score (Likert-like scale) between 1 and 5 (1, not suspect; 2, hardly suspect; 3, ambiguous; 4, suspect; 5, highly suspect)[17]. The assigned scores of 3–5 were considered positive, and scores of 1–2 were considered negative for cancer. For patients with more than one region suspected to be cancer, only the region with the highest sum of the PIRADS scores was used for statistical analysis.

## Statistical Analysis

Outcomes of interest were: upgrading, extracapsular extension (ECE), unfavorable prognosis (occurrence of both upgrading and ECE), large tumor volume (≥0.5ml) seminal vesicle invasion (SVI). Unfavorable prognosis was also evaluated considering separately unfavorable prognosis with primary GS = 4. Informative parameters for the distribution of continuous variables (age, PSA, PSAD, prostate volume) were calculated and their distributions were tested for normality by the Kolmogorov-Smirnov test. Univariate analyses were performed to evaluate the association of patient and tumor characteristics with upgrading, ECE, unfavorable prognosis, large tumor volume and seminal vesicle invasion. The association for continuous variables was assessed by T-test or non-parametric two-sample Wilcoxon test, as appropriate; the association for categorical variables was assessed by Chi-Square test or Fisher’s Exact Test, as appropriate. Sensitivity, specificity, positive predicted values (PPV) and negative predicted values (NPV) for PIRADS score 3–5 (positive for cancer) versus 1–2 (negative for cancer) were calculated for each outcome of interest. Multivariate unconditional logistic regression models were performed to assess the independent contribution of patient and tumor characteristics in the prediction of upgrading, ECE, unfavorable prognosis, large tumor volume and seminal vesicle invasion; Odds Ratios (OR) and 95% Confidence Intervals (CI) were calculated. Receiver Operating Characteristic (ROC) curves were drawn for models with and without inclusion of PIRADS score, and the corresponding areas under the curve (AUC) of the two models were compared with the De Long test. To graphically evaluate the net benefit for the models with and without inclusion of PIRADS score, a decision-curve analysis (DCA) was performed. DCA expresses the ‘‘net benefit” of a prediction model as the difference between the proportion of patients who are true positive and the proportion who are false positive, the latter weighted by the relative harm of a false–positive and a false–negative result [18].

Statistical significance was defined as p<0.05. Statistical analysis was performed using SAS software, version 9.2. The DCA was performed by using an Excel macro (Microsoft Office Excel 2007).

## Results

Table 1 presents the main characteristics of the study population. Sensitivity for MRI in identifying tumors with the most unfavorable prognostic characteristics was extremely high, ranging from 94% for large tumor volume to 100% for cancers with ECE, unfavorable prognosis and SVI (Table 2). MRI presented an excellent ability in ruling out almost all the outcomes of interest: NPV was 94% for upgrading and 100% for ECE, unfavorable prognosis and SVI (Table 2). On the other side, specificity and PPV values were generally low for almost all the outcomes of interest, with the exception of tumor volume, for which we found a PPV = 97%, probably due, however, to the very low number of patients with tumor volume <0.5 ml (Table 2).

**Table 1:** Patient and tumor characteristics of the study population.

	N (%)
Volume^	47.94 (±14.53)
Age^	62.75 (±8.28)
Clinical Stage	
cT1c	191 (85.65%)
cT2a	32 (14.35%)
PSA^	6.02 (±1.91)
PSA Density^	0.13 (±0.04)
Tumor volume^	0.95 (±0.23)
Pathological stage	
pT2a	23 (10.31%)
pT2b	3 (1.35%)
pT2c	145 (65.02%)
pT3a	45 (20.18%)
pT3b	7 (3.14%)
Positive Cores	
1	95 (42.60%)
2	128 (57.40%)
Pathological Total Gleason Score	
6	110 (49.33%)
7	110 (49.33%)
8	2 (0.90%)
9	1 (0.45%)
Cancer at MRI	
Not visible	19 (8.52%)
Visible	204 (91.48%)
Positive lymph nodes	
Yes	4 (1.79%)
No	219 (98.21%)
Seminal vesicle invasion	
Yes	7 (3.15%)
No	215 (96.85%)
PIRADS	
1	2 (0.91%)
2	14 (6.36%)
3	58 (26.36%)
4	71 (32.27%)
5	75 (34.09%)

^mean (± SD)

**Table 2:** Sensitivity (SE), specificity (SP), positive predicted values (PPV) and negative predicted values (NPV) with 95%(CI) for 1–2 vs. ≥ 3 PIRADS score.

	SE(CI)*	SP(CI)*	PPV(CI)*	NPV(CI)*
Upgrading	99 (95–100)	14 (8–22)	55 (48–62)	94 (70–100)
Extra capsular extension	100 (93–100)	10 (6–15)	25 (20–32)	100 (79–100)
Unfavorable prognosis	100 (91–100)	9 (5–14)	19 (14–25)	100 (79–100)
Tumor volume	94 (90–97)	40 (12–74)	97 (94–99)	25 (7–52)
Seminal vesicle invasion	100 (59–100)	8 (4–12)	3 (1–7)	100 (79–100)

*Percentage

At univariate analysis (Tables 3–7) we found a significant association between PIRADS score and GS upgrading, ECE, unfavorable prognosis and large tumor volume: the probability of each outcome of interest increased with increasing PIRADS score (p<0.0001).The same trend was confirmed when restricting the analysis to patients with unfavorable prognosis and primary GS = 4 (p = 0.01). No significant association was found between PIRADS score and SVI (p = 0.28), although a significant trend for one–unit increase in PIRADS score was observed even for this outcome (p = 0.03). Other possible predictors of unfavorable prognostic characteristics were: age (upgrading, unfavorable prognosis), clinical stage (ECE, unfavorable prognosis, SVI), PSA and PSA density (unfavorable prognosis).

**Table 3:** Association of patient and tumor characteristics withupgrading: univariate and multivariate analysis.

	Upgrading		Pvalue*	Multivariate Odds Ratio (95%CI)	Pvalue
	“Yes” N (%)	“No” N (%)			
Volume^	47.55 (±10.25)	48.34 (±17.94)	0.43	1.00 (0.98–1.03)^2^	0.92
Age^	63.35 (±9.38)	62.13 (±6.96)	0.04	1.01 (0.98–1.05)^2^	0.53
Clinical Stage			0.34		0.59
cT1c	94 (83%)	97 (88%)		1.00 (reference)	
cT2a	19 (17%)	13 (12%)		1.27 (0.54–2.97)	
PSA^	6.09 (±1.95)	5.94 (±1.87)	0.56	1.02 (0.86–1.19)^2^	0.85
PSA Density^	0.13 (±0.04)	0.13 (±0.04)	0.76	-^3^	-
Positive Cores			0.28		0.55
1	44 (39%)	51 (46%)		1.00 (reference)	
2	69 (61%)	59 (54%)		1.20 (0.66–2.18)	
PIRADS			<0.0001^1^	2.72 (1.93–3.84)^2^	<0.0001
1	0 (0%)	2 (2%)			
2	1 (1%)	13 (12%)			
3	17 (15%)	41 (38%)			
4	40 (35%)	31 (29%)			
5	55 (49%)	20 (19%)			
Cancer at MRI			<0.0001	-^3^	-
Not visible	1 (1%)	18 (16%)			
Visible	112 (99%)	92 (84%)			

^1^Mantel-Haenszel p-value for trend = <0.0001

*T test or non parametric two-sample Wilcoxon test for continuous variables, as appropriate; Chi-Square test or Fisher’s Exact Test for categorical variables, as appropriate

^2^One-unit increase OR

^3^Not entered in the multivariate model because it is a linear combination of other variables.

Note: significant ORs and p-values are in bold.

**Table 4:** Association of patient and tumor characteristics with extra capsular extension: univariate and multivariate analysis.

	Extra capsular extension		Pvalue*	Multivariate Odds Ratio (95%CI)	Pvalue
	“Yes” N (%)	“No” N (%)			
Volume^	47.77 (±10.10)	47.99 (±15.66)	0.54	0.99 (0.96–1.03)^2^	0.73
Age^	63.85 (±8.17)	62.41 (±8.31)	0.17	0.99 (0.95–1.04)^2^	0.79
Clinical Stage			0.02		0.02
cT1c	39 (75%)	152 (89%)		1.00 (reference)	
cT2a	13 (25%)	19 (11%)		3.19 (1.22–8.35)	
PSA^	6.56 (±2.23)	5.86 (±1.78)	0.07	1.27 (1.03–1.57)^2^	0.03
PSA Density^	0.14 (±0.04)	0.13 (±0.04)	0.10	-^3^	-
Positive Cores			0.34		0.68
1	19 (37%)	76 (44%)		1.00 (reference)	
2	33 (63%)	95 (56%)		1.17 (0.55–2.49)	
PIRADS			<0.0001^1^	5.27 (2.94–9.44)^2^	<0.0001
1	0 (0%)	2 (1%)			
2	0 (0%)	14 (8%)			
3	1 (2%)	57 (34%)			
4	15 (29%)	56 (34%)			
5	36 (69%)	39 (23%)			
Cancer at MRI			0.01	-^3^	-
Not visible	0 (0%)	19 (11%)			
Visible	52 (100%)	152 (89%)			

^1^Mantel-Haenszel p-value for trend = <0.0001

*T test or non parametric two-sample Wilcoxon test for continuous variables, as appropriate; Chi-Square test or Fisher’s Exact Test for categorical variables, as appropriate

^2^One-unit increase OR

^3^Not entered in the multivariate model because it is a linear combination of other variables.

Note: significant ORs and p-values are in bold.

**Table 5:** Association of patient and tumor characteristics with unfavorable prognosis: univariate and multivariate analysis.

	Unfavorable prognosis N (%)	Favorable prognosis N (%)	Pvalue*	Multivariate Odds Ratio (95%CI)	Pvalue
Volume^	49.08 (±10.01)	47.70 (±15.33)	0.20	1.01 (0.97–1.05)^2^	0.74
Age^	65.36 (±7.80)	62.19 (±8.29)	0.01	1.04 (0.98–1.10)^2^	0.26
Clinical Stage			0.03		0.04
cT1c	29 (74%)	162 (88%)		1.00 (reference)	
cT2a	10 (26%)	22 (12%)		2.96 (1.06–8.22)	
PSA^	6.87 (±2.08)	5.84 (±1.83)	0.002	1.36 (1.08–1.72)^2^	0.01
PSA Density^	0.14 (±0.04)	0.13 (±0.04)	0.04	-^3^	-
Positive Cores			0.38		0.98
1	14 (36%)	81 (44%)		1.00 (reference)	
2	25 (67%)	103 (56%)		1.01 (0.43–2.37)	
PIRADS			<0.0001^1^	5.42 (2.74–10.70)^2^	<0.0001
1	0 (0%)	2 (1%)			
2	0 (0%)	14 (8%)			
3	0 (0%)	58 (32%)			
4	11 (28%)	60 (33%)			
5	28 (72%)	47 (26%)			
Cancer at MRI			0.05	-^3^	-
Not visible	0 (0%)	19 (10%)			
Visible	39 (100%)	165 (90%)			

^1^Mantel-Haenszel p-value for trend = <0.0001

*T test or non parametric two-sample Wilcoxon test for continuous variables, as appropriate; Chi-Square test or Fisher’s Exact Test for categorical variables, as appropriate

^2^One-unit increase OR

^3^Not entered in the multivariate model because it is a linear combination of other variables.

Note: significant ORs and p-values are in bold.

**Table 6:** Association of patient and tumor characteristics with tumor volume: univariate and multivariate analysis.

		Tumor volume		Pvalue*	Multivariate Odds Ratio (95%CI)	Pvalue
		≥0.5 ml N (%)	<0.5 ml N (%)			
	Volume^	47.52 (±11.25)	55.25 (±42.24)	0.30	0.99 (0.96–1.01)^2^	0.31
	Age^	62.66 (±8.39)	64.27 (±5.96)	0.53	0.97 (0.87–1.08)^2^	0.58
	Clinical Stage			0.68		0.47
	cT1c	181 (86%)	10 (83%)		1.00 (reference)	
	cT2a	30 (14%)	2 (17%)		0.49 (0.07–3.42)	
	PSA^	6.05 (±1.92)	5.41 (±1.68)	0.26	1.33 (0.89–1.97)^2^	0.17
	PSA Density^	0.13 (±0.04)	0.12 (±0.05)	0.39	-^3^	-
	Positive Cores			0.26		0.62
	1	88 (42%)	7 (58%)		1.00 (reference)	
	2	123 (58%)	5 (42%)		1.41 (0.37–5.43)	
	PIRADS			<0.0001^1^	3.43 (1.56–7.65)^2^	0.002
	1	2 (1%)	0 (0%)			
	2	10 (5%)	4 (40%)			
	3	53 (25%)	5 (50%)			
	4	71 (34%)	0 (0%)			
	5	74 (35%)	1 (10%)			
	Cancer at MRI			<0.0001	-^3^	-
	Not visible	13 (6%)	6 (50%)			
	Visible	198 (94%)	6 (50%)			

^1^Mantel-Haenszel p-value for trend = 0.0002

*T test or non parametric two-sample Wilcoxon test for continuous variables, as appropriate; Chi-Square test or Fisher’s Exact Test for categorical variables, as appropriate

^2^One-unit increase OR

^3^Not entered in the multivariate model because it is a linear combination of other variables.

Note: significant ORs and p-values are in bold.

**Table 7:** Association of patient and tumor characteristics with seminal vesicle invasion: univariate and multivariate analysis.

	Seminal vesicle invasion		Pvalue*	Multivariate Odds Ratio (95%CI)	Pvalue
	“Yes” (%)	“No” (%)			
Volume^	46.14 (±3.72)	47.94 (±14.76)	0.98	1.01 (0.92–1.10) ^2^	0.91
Age^	66.57 (±5.70)	62.63 (±8.35)	0.17	1.06 (0.92–1.21) ^2^	0.44
Clinical Stage			0.01		0.02
cT1c	3 (43%)	187 (87%)		1.00 (reference)	
cT2a	4 (57%)	28 (13%)		7.78 (1.44–41.93)	
PSA^	6.04 (±1.91)	6.01 (±1.91)	0.99	1.01 (0.63–1.61) ^2^	0.98
PSA Density^	0.13 (±0.04)	0.13 (±0.04)	0.89	-^3^	-
Positive Cores			0.70		0.88
1	2 (29%)	92 (43%)		1.00 (reference)	
2	5 (71%)	123 (57%)		1.15 (0.19–6.98)	
PIRADS			0.28^1^	3.97 (0.92–17.09) ^2^	0.06
1	0 (0%)	2 (1%)			
2	0 (0%)	14 (7%)			
3	0 (0%)	58 (27%)			
4	2 (29%)	68 (32%)			
5	5 (71%)	70 (33%)			
Cancer at MRI			1.00	-^3^	-
Not visible	0 (0%)	19 (9%)			
Visible	7 (100%)	196 (91%)			

^mean (± SD)

*T test or non parametric two-sample Wilcoxon test for continuous variables, as appropriate; Chi-Square test or Fisher’s Exact Test for categorical variables, as appropriate

^1^Mantel-Haenszel p-value for trend = 0.03

^2^ One-unit increase OR

^3^ Not entered in the multivariate model because it is a linear combination of other variables.

Note: significant ORs and p-values are in bold.

At multivariate analysis (Table 3) the association of PIRADS score with upgrading, ECE, unfavorable prognosis and large tumor volume was confirmed. The risk of having unfavorable prognosis was more than quintupled for every unit increase of PIRADS score. Clinical stage cT2a was a significant independent predictor of ECE, unfavorable prognosis and SVI, while PSA was a significant independent predictor of ECE and unfavorable prognosis.

**Fig 1:**
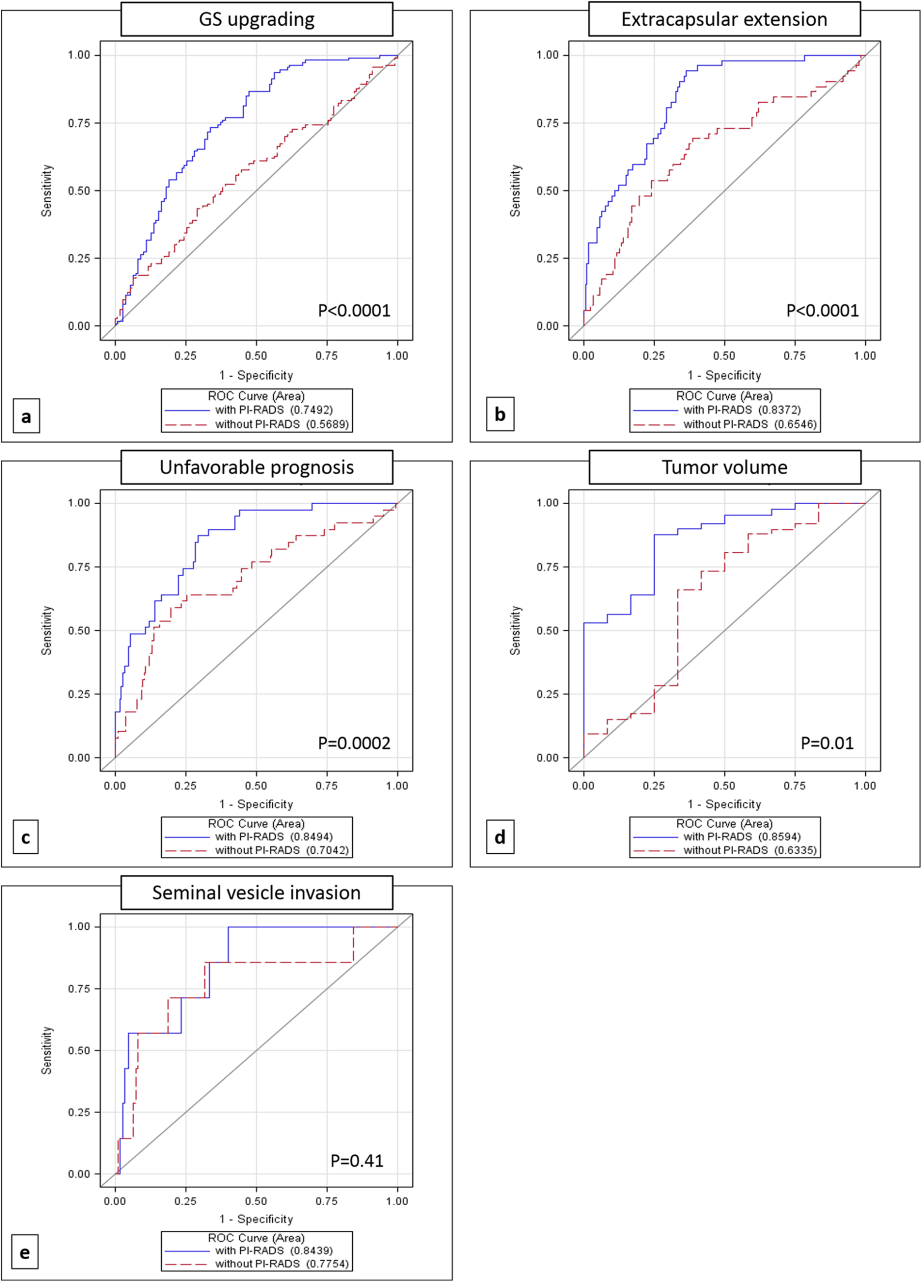
ROC Curves comparing models with and without inclusion of PIRADS score for a) Gleason score (GS) upgrading, b) extra capsular extension, c) unfavorable prognosis, d) tumor volume and e) seminal vesicle invasion.

Fig 1 shows the ROC curves comparing models with and without PIRADS score. The differences between the correspondent AUC were statistically significant for upgrading (p<0.0001), ECE (p<0.0001), unfavorable prognosis (p = 0.0002), and tumor volume (p = 0.01), whereas it was not significant for SVI (p = 0.41) probably due to the very low number of patients with SVI.

**Fig 2:**
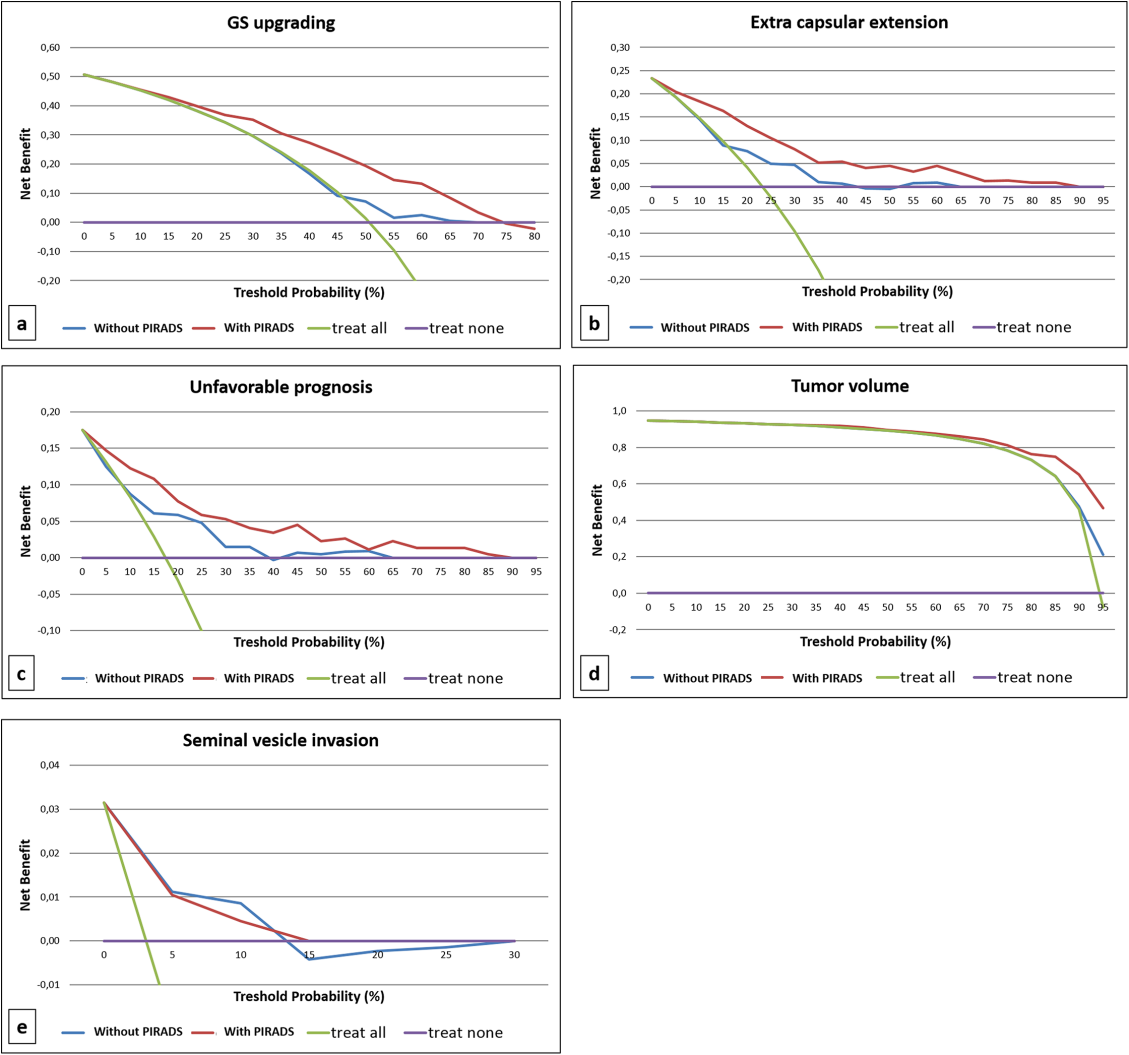
Decision curve analysis of the effect of prediction models on the detection of a) upgrading, b) extra capsular extension, c) unfavorable prognosis, d) tumor volume and e) seminal vesicle invasion. Model with PIRADS score (red line) is plotted against treat none (violet line), treat all (green line) and model without PIRADS score (blue line).

Fig 2 presents the decision curves for the multivariable models presented in Table 2 and Fig 1. Models including PIRADS score resulted in greater net benefit for almost all the outcomes of interest if compared with models without the inclusion of PIRADS score, again with the only exception of SVI. Inclusion of PIRADS score in prediction tools may therefore increase the net benefit over almost all the range of probabilities when the outcome of interest is upgrading, upstaging or their combination (unfavorable prognosis), while it results in increased net benefit only at a threshold probability>80% when the outcome of interest is tumor volume.

## Discussion

The proportion of men with low-risk PCa ranged from 16% in 2000 to 21% in 2006, showing an increasing of 'watchful waiting option' from 0% to 39% over the same period[19]. These data confirm the favorable outcomes of watchful waiting reported in the PIVOT study[20]. Thus the goal of PCa care is to identify and treat only men with clinically significant disease. In this setting, AS aims to avoid unnecessary treatment in men with slow-growing PCa, although current risk stratification schemes misclassify some patients. Selvadurai et al observed that about one-third of those men undergoing deferred RP had adverse features at the time of surgery, such as extracapsular extension, high-grade disease, or positive margins[21]. Circulating biomarkers represent a promising approach to identify men with apparently low-risk biopsy pathology, but who harbor potentially aggressive tumors unsuitable for AS[22,23]. Recently van den Bergh et al. provided a summary of the current studies examining imaging and novel biomarkers in AS for PCa, emphasizing their burden role of monitoring during AS[4]. Several studies have suggested the benefit of early repeat biopsy or more extended biopsy to reduce the risk of unfavorable disease on RP specimens regardless of how AS criteria are defined[24,25]. Kuru et al in a retrospective evaluation of the PIRADS in mpMRI based on single cores and single-core histology, confirmed a significant correlation between this decision-support scoring system and histopathology[26]. The adding performance of MRI to the initial clinical evaluation of men with clinically low risk PCa helped prediction, showing that an overall PIRADS score of 5 had a high sensitivity for GS upgrading on confirmatory biopsy, and suggesting a potential role in patients’ selection for AS[27]._._Recently, Abdi et al demonstrated on multivariate analysis an increased rate of AS termination for patients with PIRADS score 4 or 5 (vs 3) undergoing MRI fusion technology during transrectal ultrasound-guided biopsy[28]. Bittencourt et al in 133 consecutive PCa patients, who underwent prostatectomy, showed moderate overall accuracy of ESUR/PIRADS criteria in the prediction of EPE in a subpopulation with intermediate to high-risk disease and large-volume tumors [29].

Other authors[30,31] showed that MRI does not improve the prediction of high-risk and/or non organ-confined disease in a RP specimen.

According to previous reports [32,33], our study supports the prognostic accuracy of MRI and PIRADS score in predicting pathological features such as GS upgrading, ECE, unfavorable prognosis and large tumor volume in a cohort of patients eligible for AS. Particularly, considering the multivariable model for predicting unfavorable prognosis, we found a strong association with one unit increase PIRADS score as well as with one unit increase PSA and clinical stage cT2a compared with cT1c. DCA further confirmed the benefit given by using a model including PIRADS score when compared with the decision of treating all patients or treating none, as well as compared with a model that do not include this scoring system. The inclusion of PIRADS score in prediction tools may increase the net benefit over almost all the range of probabilities when the outcome of interest is GS upgrading, ECE or their combination. In particular, we found that the PIRADS score for detecting cancer was highly sensitive for both ECE and seminal vesicle invasion, although we did not use PIRADS-specific scores in order to assess these variables. Also, it results in increased net benefit at a threshold probability>80% when the outcome of interest was tumor volume.

## Conclusions

Our findings show that mpMRI and PIRADS scoring are feasible tools in clinical setting and could be used as decision-support systems for a more accurate selection of patients eligible for AS. ROC curves and DCA showed the higher accuracy of the models including PIRADS score in predicting GS upgrading, ECE, unfavorable prognosis and tumor volume at final histology.
